# Distinct and Overlapping Functions of *ptpn11* Genes in Zebrafish Development

**DOI:** 10.1371/journal.pone.0094884

**Published:** 2014-04-15

**Authors:** Monica Bonetti, Virginia Rodriguez-Martinez, Jeroen Paardekooper Overman, John Overvoorde, Mark van Eekelen, Chris Jopling, Jeroen den Hertog

**Affiliations:** 1 Hubrecht Institute-Koninklijke Nederlandse Akademie van Wetenschappen and University Medical Center Utrecht, Utrecht, The Netherlands; 2 Institute of Biology, Leiden, Leiden, The Netherlands; University of Sheffield, United Kingdom

## Abstract

The *PTPN11* (protein-tyrosine phosphatase, non-receptor type 11) gene encodes SHP2, a cytoplasmic PTP that is essential for vertebrate development. Mutations in *PTPN11* are associated with Noonan and LEOPARD syndrome. Human patients with these autosomal dominant disorders display various symptoms, including short stature, craniofacial defects and heart abnormalities. We have used the zebrafish as a model to investigate the role of Shp2 in embryonic development. The zebrafish genome encodes two *ptpn11* genes, *ptpn11a* and *ptpn11b*. Here, we report that *ptpn11a* is expressed constitutively and *ptpn11b* expression is strongly upregulated during development. In addition, the products of both *ptpn11* genes, Shp2a and Shp2b, are functional. Target-selected inactivation of *ptpn11a* and *ptpn11b* revealed that double homozygous mutants are embryonic lethal at 5–6 days post fertilization (dpf). *Ptpn11a-/-ptpn11b-/-* embryos showed pleiotropic defects from 4 dpf onwards, including reduced body axis extension and craniofacial defects, which was accompanied by low levels of phosphorylated Erk at 5 dpf. Interestingly, defects in homozygous *ptpn11a-/-* mutants overlapped with defects in the double mutants albeit they were milder, whereas *ptpn11b-/- single mutants* did not show detectable developmental defects and were viable and fertile. *Ptpn11a-/-ptpn11b-/-* mutants were rescued by expression of exogenous *ptpn11a* and *ptpn11b* alike, indicating functional redundance of Shp2a and Shp2b. The *ptpn11* mutants provide a good basis for further unravelling of the function of Shp2 in vertebrate development.

## Introduction

The *PTPN11* (protein-tyrosine phosphatase, non-receptor type 11) gene encodes SHP2, a ubiquitously expressed cytoplasmic protein-tyrosine phosphatase (PTP) with two Src homology 2 (SH2) domains that is essential for normal development [1]–[3]. SHP2 is a positive effector of extracellular regulated kinase (ERK)/MAPK signal transduction, downstream of most receptor tyrosine kinases (RTKs) [2] [3]. Moreover, Shp2 is involved in other signal transduction processes as well, including Jak-STAT signaling [3] and PI3K-AKT (also known as PI3K-PKB) signaling [2].

Missense mutations in the *PTPN11* gene are associated with the dominantly inherited genetic disorders, Noonan syndrome (NS) (OMIM 163950) and LEOPARD syndrome (LS) (OMIM 151100) [4]–[9]. These syndromes share several overlapping features, including facial dysmorphism, cardiovascular defects, hearing loss, growth retardation and scoliosis [7], [10]. NS variants of SHP2 have increased PTP activity [2]. By contrast, most LS variants of SHP2 display reduced catalytic activity [11]–[14]. NS-associated mutations in Shp2 promote Erk/MAPK signaling [2]. Yet, the role of LS Shp2-variants in Erk/MAPK signaling is less clear, in that both activation and inhibition of Erk/MAPK signaling have been observed in LS [11], [14]–[18]. Nevertheless, signaling by NS- and LS-variants of SHP2 is distinct, because LS-SHP2, but not NS-SHP2, affects AKT (PKB) signaling [12], [19]. The mechanism underlying how NS- and LS-variants of SHP2 induce similar defects in development remains to be determined.

Shp2 has an essential role in early development in the mouse [20]. Homozygous *Ptpn11*-/- embryos die pre-implantation due to defective Erk activation and trophoblast stem cell death [20], precluding the possibility to study Shp2 function in differentiated cell types in adult animals. This problem was partially overcome with the use of conditional Shp2 knockout mice [21]–[23]. For example, mice with a *Ptpn11* deletion in the cardiomyocytes show that Shp2 is required for the suppression of dilated cardiomyopathy [22]. Conditional deletion of Shp2 in neural progenitor cells results in early postnatal lethality, impaired corticogenesis, and reduced proliferation of progenitor cells in the ventricular zone [21]. In an aged mouse model, hepatocyte-specific deletion of *Ptpn11* promotes inflammatory signalling and hepatic inflammation/necrosis, resulting in regenerative hyperplasia and spontaneous development of tumors [24]. Furthermore, a study in *Xenopus* shows that dominant-negative Shp2 mutants failed to complete gastrulation, resulting in severe posterior truncations [25]. In zebrafish, Shp2 knockdown embryos show a reduction of the body axis extension at 10 hours post fertilization (hpf) that is consistent with gastrulation defects. In addition, at later stages, the embryos are shorter and develop a hammerhead phenotype [26], [27]. These data show that Shp2 is a crucial player in the development of many different organisms.

In our study we used zebrafish as a model to further investigate the role of Shp2. The zebrafish has proven to be an excellent experimental model to study gene function *in vivo* and to analyze vertebrate embryogenesis as development occurs externally and the embryos are transparent [28]. We identified two zebrafish *ptpn11* genes (*ptpn11a* and *ptpn11b*), encoding Shp2a and Shp2b, respectively. We demonstrate that *ptpn11a* was constitutively expressed and *ptpn11b* expression increased during early embryogenesis. *Ptpn11a* and *ptpn11b* both encoded functional proteins with similar catalytic activity. We used target selected gene inactivation (TSGI) to identify stop mutations in the N-terminal SH2 domain of each of the two *ptpn11* genes. Our study showed that *ptpn11b-/-* zebrafish were viable and fertile and showed no abnormal phenotype during development. However, single homozygous mutants for *ptpn11a* and double homozygous mutant embryos exhibited abnormalities such as craniofacial defects and reduced length, and these mutants died at 5 days post fertilization (dpf). Moreover, we found that Shp2 deletion suppressed Erk activation in double mutant embryos, but not in embryos lacking only Shp2a or Shp2b. Together, our observations demonstrate that Shp2 is essential for zebrafish development, and that *ptpn11a* has a more pronounced role as the result of its higher expression level at early stages.

## Materials and Methods

### Ethics statement

All procedures involving experimental animals described here were approved by the local animal experiments committee (KNAW-DEC protocol HL05.1501) and performed according to local guidelines and policies in compliance with national and European law.

### Zebrafish care and generation of *ptpn11* mutant lines

Zebrafish maintenance, breeding and staging were performed following published protocols [29]. ENU mutagenesis was performed on TL males [30] and exons 1–4 of the zebrafish *ptpn11a* and *ptpn11b* genes were sequenced using DNA from F1 generation zebrafish. Each mutation was confirmed by resequencing. Genomic DNA was extracted from adult fish fin clip or fixed embryos. The genotyping assay for the *ptpn11* mutations was performed by nested PCR with primer sets: *ptpn11a*, 5′-GCGCTGTCACACACATTAAGA, 5′-TCACAGCCAATAAAGAGAAGC; nested *ptpn11a* 5′-CGACCTGTATGGTGGAGAGAA, 5′-TCCCAAATTGTCATGTAAGG; *ptpn11b*, 5′- TTCAACAACATCCTCCTAACTG, 5′-AAACAAACCACAGCTCTTCC and nested *ptpn11b* 5′-GTCTGTCATCCCTCATTTCC, 5′-GCAGGATTTATTCTGTCCAC, followed by sequencing to detect the mutations.

### RNA isolation, cDNA synthesis and quantitative PCR

For the reverse transcription PCR (RT-PCR) experiment, thirty zebrafish embryos at various stages of development (4 hpf, 10 hpf, 1 dpf, 2 dpf, 3 dpf and 5 dpf) were collected and homogenized in TRIzol Reagent (Invitrogen; Carlsbad, CA). The homogenate was centrifuged (12000 g, 4°C, 10 min.). 0.2 ml of Chloroform were added to the supernatant, vortexed for 15 sec., incubated for 3 min. at room temperature (RT) and then centrifuged (12000 g, 4°C, 15 min.). 0.5 ml isopropanol were added to the upper phase, incubated for 10 min. at RT and centrifuged (12000 g, 4°C, 15 min.). The pellet was washed with 75% EtOH and after vortexing, centrifuged (10000 g, 4°C, 5 min.). The pellet was then dried for 5 min. and dissolved in 100 µl MQ. After, the solution was incubated for 10 min. at 37°C, precipitated in 250 µl 100% EtOH and 10 µl 3 M NaAc and stored at −80°C.

For RT-PCR with M-MLV-RT (Promega) 1 µg of total RNA was used. PCR of cDNA was performed using the standard protocol of GoTaq (Promega) using oligonucleotides listed as follow; *ptpn11a* forward: 5′- ATGTGCCCAAGACTATCCAGATG, *ptpn11a* reverse: 5′- CCCACGTTCTCATAGACTCGAGA, *ptpn11b* forward: 5′- ACTGTGACATTGACATCCCAAAAA, *ptpn11b* reverse: TACTGCTGGTGGAGCCCTTTGGAT. The same primers were used for sequencing of the DNA fragments.

Real-time RT-PCR was performed using the IQ SYBR Green Supermix (Biorad). Real time PCR MyIQ software (Bio-rad) was used to determine the amplification cycle in which product accumulation was above the threshold cycle values (Ct). PCR was carried out after incubation at 50°C for 2 min and pre-denaturing at 95°C for 3 min, followed by 40 cycles at 95°C for 30 sec and 62°C for 1 min. The relative quantification was given by the CT values, determined by triplicate reactions for all of the samples for *ptpn11a*, *ptpn11b* and *β-actin*. Real time PCR Ct values were analyzed using the 2−ΔCT method [31].

For the quantification of *ptpn11a* and *ptpn11b* mRNA levels, the housekeeping gene actin was used as internal standard. ΔCT-values were calculated as follows: CT(*β-actin*)-CT(*ptpn11*) and were normalized to the starting point T0 (4 hpf).

### *In situ* hybridization

*In situ* hybridizations were done essentially as described [32]. Digoxigenin-UTP-labeled riboprobes for *ptpn11a* and *ptpn11b* were synthesized from PCR products. The PCR products were amplified with primers containing the T7 promoter and then sequenced. Primers were designed as follows: *ptpn11a* forward, 5′- ATTTAGGTGACACTATAGGGAGCTACATTGCCACACAA, *ptpn11a* reverse: 5′- TAATACGACTCACTATAGGGTTTTTCATCTCTCGGTTTAGTCA. For *ptpn11b*, primers that attached to the 3′UTR region of the gene were designed: *ptpn11b* forward: 5′- ATTTAGGTGACACTATAGGGAACAAAACGAAGGAGAGG, *ptpn11b* reverse: 5′- TAATACGACTCACTATAGGGGCCTGCCTCATTTTTAGCTG. Embryos were cleared in methanol and mounted in Benzyl Benzoate/Benzyl Alcohol (2∶1) before pictures were taken.

### Alcian blue staining

Morphological phenotypes were assessed at 5 dpf. Embryos were anesthesized at 5 dpf with MS-222 (Sigma), fixed in 4% paraformaldehyde (PFA) and the cartilage was stained with alcian blue. Briefly, fixed embryos were washed for 10 min in 50% ETOH in water. Next, the embryos were stained overnight at 4C in staining solution (0.04% alcian blue, 50 mM MgCl2, 70% EtOH). Embryos were washed in 0.2% of Triton X-100 in water. Pictures of lateral and ventral views were taken. The width of the ceratohyal angle was determined using Image J software and the ratio was determined as a direct measure for craniofacial defects. Averages were determined and a student *t*-test was done to determine whether the differences between the diverse conditions were statistically significant.

### Immunoblotting

Zebrafish embryos were raised in standard conditions. At 5 dpf, 5 embryos were pooled and lysed in buffer containing 50 mM Tris, pH 7.5, 150 mM NaCl, 1 mM EDTA, 1 mM sodium orthovanadate, 1% Nonidet P-40, 0.1% sodium deoxycholate, protease inhibitor mixture (Complete Mini, Roche Diagnostics) and vanadate, and 40 µl lysisbuffer. Lysates were spun down and 4× sample buffer was added to supernatant; Samples were run on SDS-PAGE gel (10%) and transferred to PVDF membrane. After transfer the membrane was stained with Coomassie Blue stain to verify equal loading of the lysates. Subsequently the PVDF membrane was blocked with 5% BSA and then incubated with the corresponding antibodies targeting mouse monoclonal antibody anti-pERK, rabbit monoclonal antibody anti-ERK, rabbit monoclonal antibody pAKT and rabbit monoclonal antibody anti-AKT. All the primary antibodies were diluted 1∶1000 in blocking solution and they were purchased from Cell-Signaling. The secondary antibodies (mouse and rabbit) conjugated to horseradish peroxidase (HRP), were purchased from BD bioscience Pharmaceutical and they were diluted 1∶10,000 in TBST. The membranes were subjected to detection by enhanced chemiluminescence (Thermo Scientific kit).

### Phosphatase assay

We generated zebrafish *ptpn11a* and *ptpn11b* cDNA constructs and cloned them in a pGEX-4t vector, allowing production and purification of recombinant Shp2 proteins, fused to six histidine residues in their C-termini. Purified GST-fusion proteins were directly incubated in PTP assay buffer (20 mM MES buffer pH 6.0, 1 mM EDTA, 150 mM NaCl, 1 mM dithiotreitol, and 10 mM p-nitrophenylphosphate) for 45 min. at 30 °C. The reactions were quenched with 0.4 M NaOH, and optical density was measured with a spectrophotometer at 415 nm (wavelength).

### Statistical analysis

Statistical significance was assessed by two-tailed Student's *t*-test in all experiments. Significance is represented in the graphs. Results are considered significant when *p*<0.05 and are expressed as mean ± standard error of the mean. * indicates a *p* value of <0.05, ** indicates a *p* value of <0.01 and *** indicates a *p* value of <0.001.

## Results

### Characterization of zebrafish *ptpn11* genes

**Figure 1:**
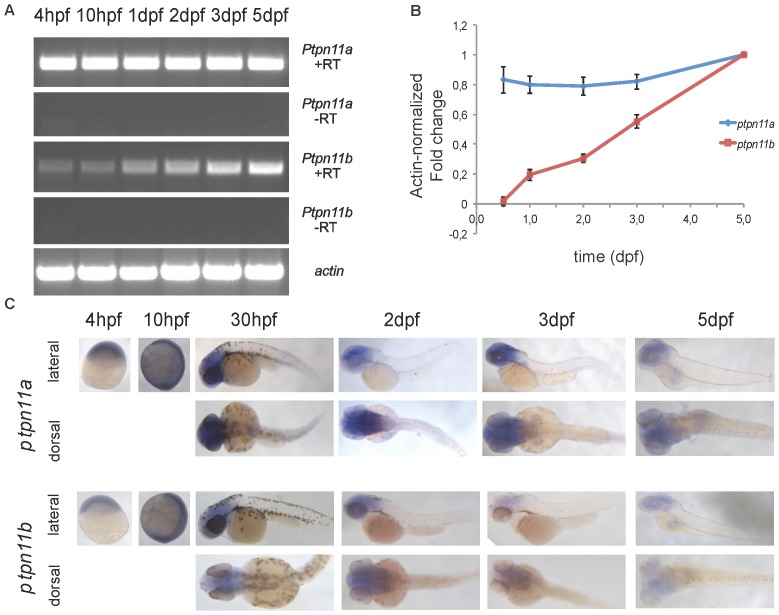
Expression of zebrafish *ptpn11a* and *ptpn11b* mRNA during embryogenesis. (a) RNA was isolated from zebrafish embryos at 4 hpf, 10 hpf, 1 dpf, 2 dpf, 3 dpf and 5 dpf. Reverse transcription PCR was performed to detect the indicated genes; reverse transcriptase was omitted from the reaction as a control (-RT). (b) Quantitative PCR for *ptpn11a* and *ptpn11b* gene expression was performed from a pool of wild-type embryos (*n* = 30) collected at 10 hpf, 1 dpf, 2 dpf, 3 dpf and 5 dpf. The values were normalized using Actin as a control and relative expression is depicted here (value at 5 dpf set to 1.0). Error bars indicate standard error of the mean. (c) Whole-mount *in situ* hybridization using *ptpn11a* and *ptpn11b* specific probes of 4 hpf, 10 hpf, 30 hpf, 2 dpf, 3 dpf and 5 dpf embryos. Lateral and dorsal views are given as indicated.

The zebrafish genome encodes two *ptpn11* genes: *ptpn11a* and *ptpn11b* [33]. The homology between human Shp2 and zebrafish Shp2a and Shp2b, encoded by *ptpn11a* and *ptpn11b* genes, is 91% and 64% respectively (Fig. S1). Due to the genome duplication that occurred in teleosts it is not uncommon for zebrafish to have two homologous copies of a gene that is present as a single copy in other vertebrates [34], [35]. To analyze stage-specific expression of *ptpn11a* and *ptpn11b* mRNA in wild-type (WT) embryos, we used a reverse transcription PCR (RT-PCR) approach. Expression levels were analysed at 4 hpf, 10 hpf, 1 dpf, 2 dpf, 3 dpf and 5 dpf. *Ptpn11a* and *ptpn11b* mRNA are both expressed from 4 hpf to 5 dpf (Fig. 1A). *Ptpn11a* mRNA was expressed at similar levels at all stages. By contrast, *ptpn11b* mRNA was hardly detectable at early stages and *ptpn11b* expression increased during development. To confirm this result, *ptpn11a* and *ptpn11b* expression levels were examined at 10 hpf, 1 dpf, 2 dpf, 3 dpf and 5 dpf by quantitative PCR. *Ptpn11a* expression was constantly maintained in all the stages whereas *ptpn11b* gene was expressed at low levels at early stages and became strongly upregulated over time (Fig. 1B). *In situ* hybridization experiments showed that the two zebrafish *ptpn11* genes were broadly expressed throughout embryonic development (Fig. 1C). Both *ptpn11* genes were maternally expressed and later in development the expression was restricted to the more anterior region of the developing embryo. Overall, these results show that both *ptpn11* genes were expressed throughout developing zebrafish embryos. *Ptpn11a* appears to be expressed at a constant level, whereas *ptpn11b* expression is low initially and is strongly upregulated over time.

### Zebrafish Shp2a and Shp2b displayed phosphatase activity

**Figure 2:**
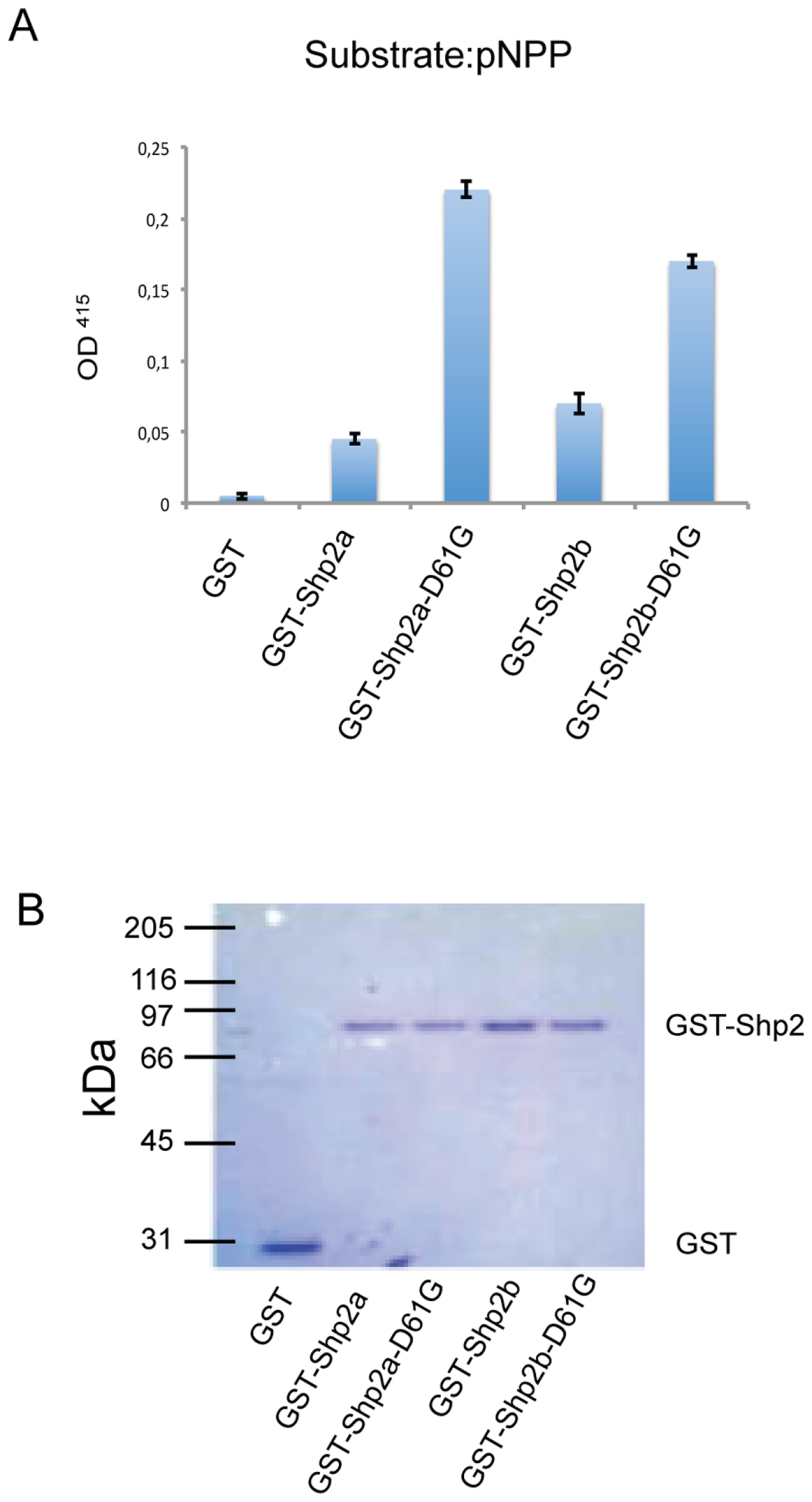
Shp2a and Shp2b proteins displayed phosphatase activity. (a) PTP activity of pGEX, WT-Shp2a, Shp2a-D61G, WT-Shp2b and Shp2b-D61G was assayed using *p*-nitrophenylphosphate (*p*NPP) and quantified spectrophotometrically. Approximately 1 µg purified fusion protein was used in the assay. Experiments were done in quadruplicate; averages are depicted and error bars indicate standard error of the mean. (b) Equivalent amounts of fusion protein that were used in the activity assays in (a) were run on an SDS-PAGE gel and stained with Coomassie blue.

Previously, we assessed that Shp2a is an active phosphatase and that mutant Shp2a-D61G with a mutation that was identified in human NS patients shows an increased phosphatase activity compared to wild type Shp2a [26]. To investigate if the *ptpn11b* gene product was functional, we expressed Shp2b in bacteria in parallel with Shp2a and investigated phosphatase activity. Both Shp2a and Shp2b displayed phosphatase activity and dephosphorylated *p*-nitrophenylphosphate (*p*NPP) to a similar extent. Moreover, Shp2b-D61G showed an increase in activity compared to WT-Shp2b, resembling the Shp2a-D61G mutant (Fig. 2). These results demonstrate that both Shp2 proteins encoded by the zebrafish genome were functional PTPs *in vitro.*

### Generation of *ptpn11* knock-out zebrafish

**Figure 3:**
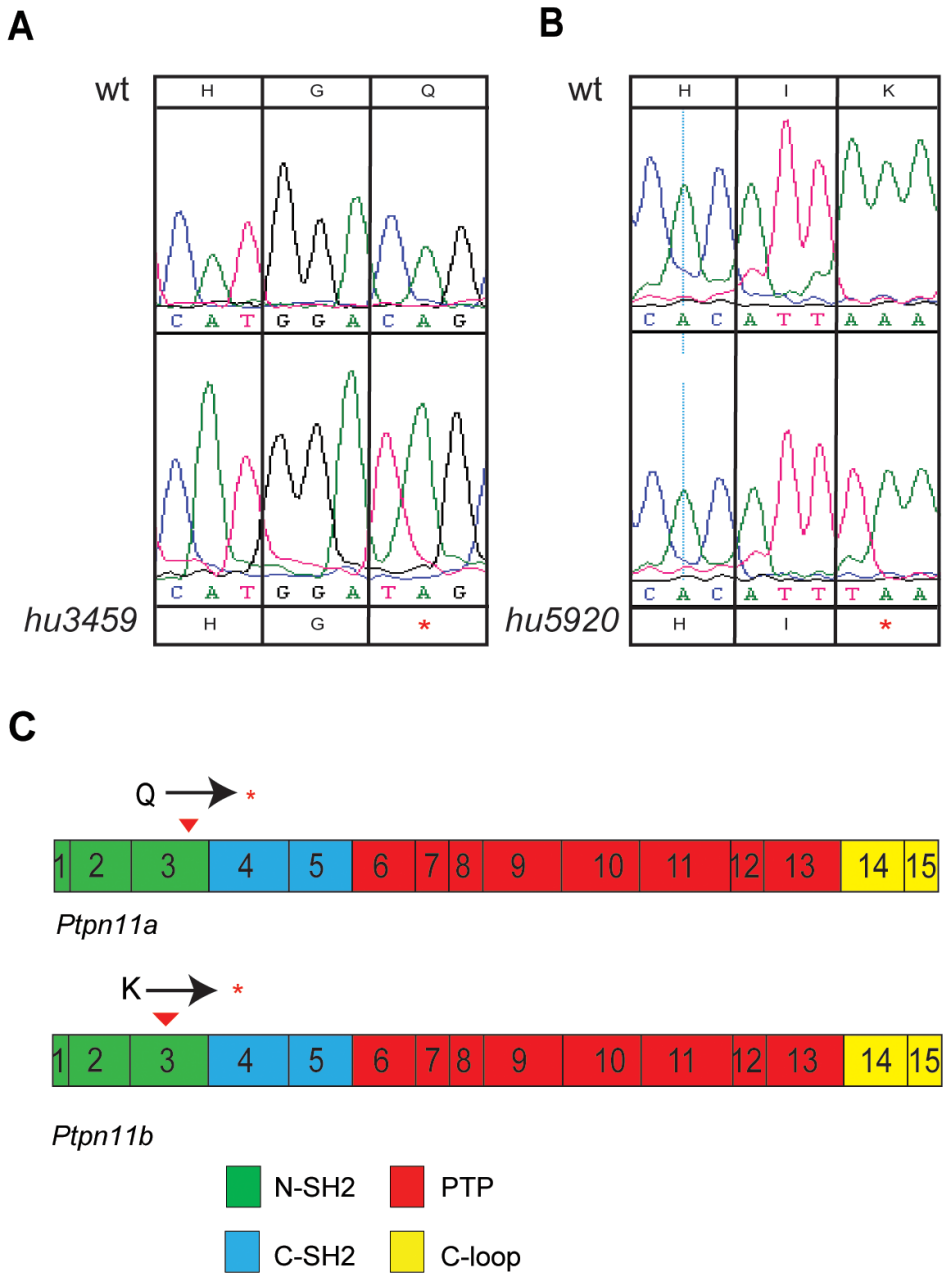
Nonsense mutations in zebrafish *ptpn11a* and *ptpn11b*. Sequence analysis of homozygous *ptpn11a* and *ptpn11b* mutants. (a) A C to T mutation in *ptpn11ahu3459* resulted in a glutamine to a STOP mutation. (b) A T to A mutation in *ptpn11bhu5920* changed lysine to a STOP mutation. (c) Schematic representation of the exon organization and structural domains of *ptpn11a* and *ptpn11b*. Nonsense mutations upstream of the catalytic domain are represented by red arrows.

**Figure 4:**
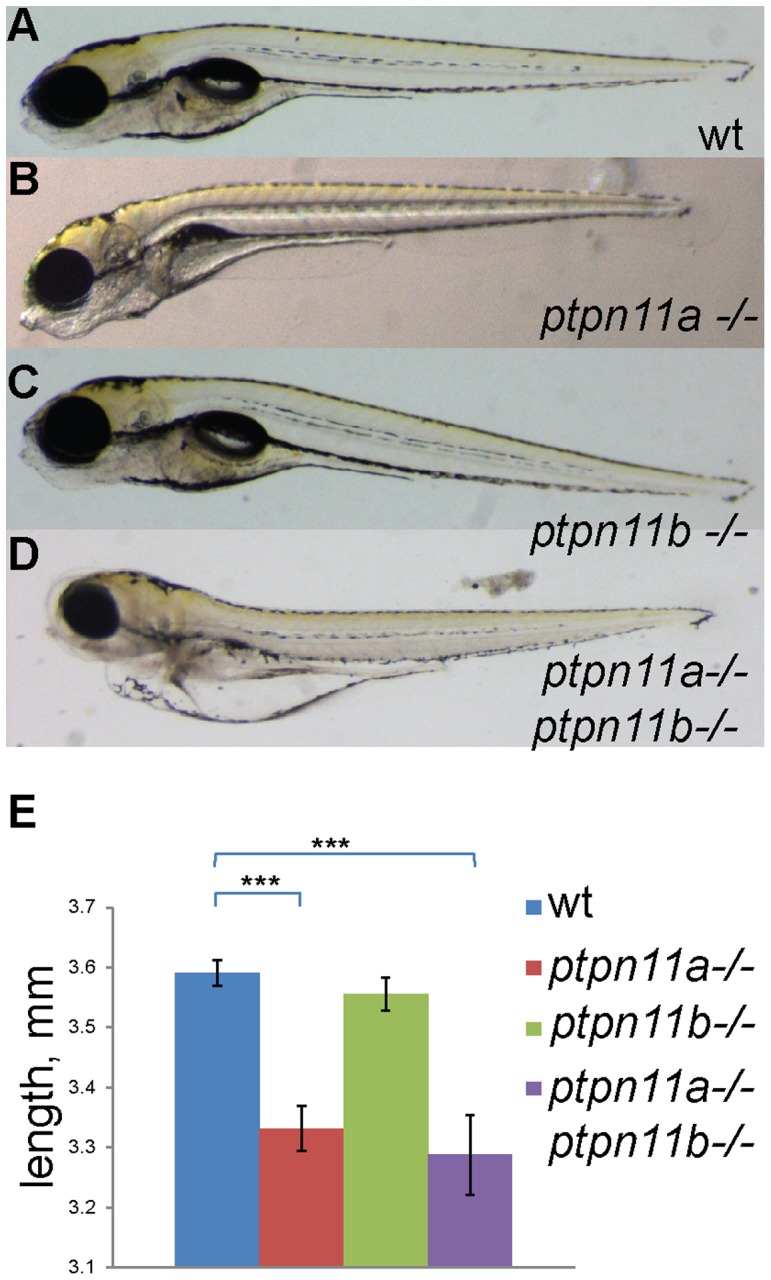
Loss of Shp2 causes severe morphological defects at 5 dpf. Morphology of 5(a) Representative wild-type, (b) *ptpn11a-/-*, (c) *ptpn11b-/-* and (d) double homozygous mutant are depicted. (e) The length was measured from nose to tip of tail at the same magnification. Bars show average length of the studied genotypes (*n* = 10-20). Statistics were determined using a student's t-test; * indicates a p value <0.05.

We identified germline mutations in the zebrafish *ptpn11* genes, using the target-selected gene inactivation (TSGI) strategy, developed at the Hubrecht Institute [30]. Mutant alleles *ptpn11ahu3459* and *ptpn11bhu5920* contained nonsense mutations in exon 3 of *ptpn11a* and *ptpn11b*, respectively (Fig. 3). We denoted the homozygous mutants as *ptpn11a*-/- and *ptpn11b*-/- because the premature stops are well upstream of the phosphatase catalytic site and hence, no functional protein is produced. *Ptpn11a*+/- and *ptpn11b*+/- fish were viable and fertile and these fish were incrossed to generate homozygous fish. Genotypes were obtained at mendelian ratios until 5 dpf. *Ptpn11a-/-* fish did not reach adulthood. By contrast, homozygous *ptpn11b-/-* fish were viable and fertile. Next, fifth generation *ptpn11a*+/- and *ptpn11b*+/- fish were incrossed and double heterozygous *ptpn11a+/-ptpn11b+/-* fish were selected. Incrosses were used to investigate zebrafish development in double homozygous *ptpn11a-/-ptpn11b-/-* embryos. Phenotypically, all genotypes were indistinguishable from wild type embryos up to 3 dpf (data not shown). However, at 5dpf, *ptpn11a*-/- and double mutant embryos displayed significant phenotypic changes such as reduced body length, malformed head, absence of swim bladder, heart edema and eye edema (Fig. 4A-D). Furthermore, these embryos were unable to move, even after stimulation. *Ptpn11b-/-* embryos were indistinguishable from wild type embryos at 5 dpf and did not display morphological defects (Fig. 4A,C; Table 1). *Ptpn11a-/-* embryos *and ptpn11a-/-ptpn11b-/-* embryos were embryonic lethal. Whereas the developmental defects of *ptpn11a-/-* and *ptpn11a-/-ptpn11b-/-* embryos were overlapping (Fig. 4B,D), the phenotype of double mutant embryos was more severe than that of *ptpn11a-/-* embryos.

**Table 1:** Characteristic features of *ptpn11a-/-*, *ptpn11b -/-* and double mutant embryos.

Characteristics	*ptpn11a-/-*	*ptpn11b-/-*	*ptpn11a-/-ptpn11b-/-*
**Head**	Reduced in some cases	Normal	Enlarged by edema. Prominent jaw
**Heart**	Normal	Normal	Severe edema
**Swim bladder**	Absent	Normal	Absent
**Length**	Shorter	Normal	Shorter
**Ventral region**	Reduced, skinny appearance	Normal	Grossly edematous. Reduced organs.

*Ptpn11a-/-* and double mutants appeared shorter than wild type embryos and we measured the body length of wildtype and mutant embryos at 5 dpf. *Ptpn11a-/-* (3.33 mm±0.03, *n* = 20) and *ptpn11a-/-ptpn11b-/-* (3.28 mm±0.05, *n* = 10) embryos were significantly shorter (*p*<0,001, Student's *t*-test) than wild type embryos (3.59 mm±0.02, *n* = 21) (Fig. 4E). The length of wildtype embryos and *ptpn11b-/-* embryos (3.55 mm±0.02, *n* = 15) did not differ significantly. Collectively, our results show that *ptpn11a*, but not *ptpn11b*, is essential for normal development. Yet, *ptpn11b* appears to be functional, because the double mutant *ptpn11a-/-ptpn11b-/-* displayed more severe defects than *ptpn11a-/-* by itself.

### Craniofacial defects in *Ptpn11a-/-* and double mutant Shp2 embryos

**Figure 5:**
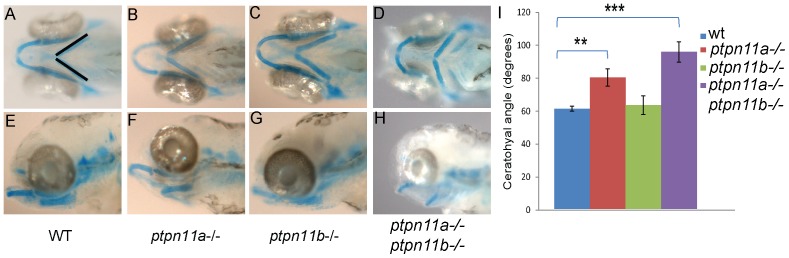
*Ptpn11a-/-* and double mutant embryos exhibit craniofacial defects at 5 dpf. Alcian blue staining was done to visualize the cartilage. (a,e) wild-type, (b,f) *ptpn11a-/-*, (c,g) *ptpn11b-/-* and (d,h) double homozygous mutant. (i) Average width of the ceratohyal angle (indicated in panel a) was determined for each genotype (*n* = 10–24). Statistics were determined using a student's t-test; ** indicates a p value of <0.01, *** indicates a p value <0.001.

Patients affected by NS and LS often suffer from craniofacial defects, including positional anomalies of the eyes and ears and a short, webbed neck. Craniofacial defects were apparent in double mutant *ptpn11a-/-ptpn11b-/-* embryos at 5 dpf. Alcian blue staining of the cartilage revealed craniofacial defects that were quantified by assessment of the angle of the ceratohyal, a direct measure for the width of the head (Fig. 5A). There was a significant difference between wildtype (60.72°±1.51, *n* = 24) and double mutant embryos (95.14°±6.12, *n* = 10) (*p*<0.001, Student's *t*-test). Moreover, also *ptpn11a-/-* embryos (79.56°±5.29, *n* = 15, *p*<0.01) had a significantly wider ceratohyal compared to wildtype embryos, although the defect was milder. *Ptpn11b-/-* embryos (62.84°±5.73, *n* = 11) did not show a significant difference compared to wildtype embryos (Fig. 5I). Taken together, these results show that particularly *ptpn11a* is indispensable for craniofacial development.

### Double mutant Shp2 phenotype was rescued upon *ptpn11a* and *ptpn11b* mRNA injections

**Figure 6:**
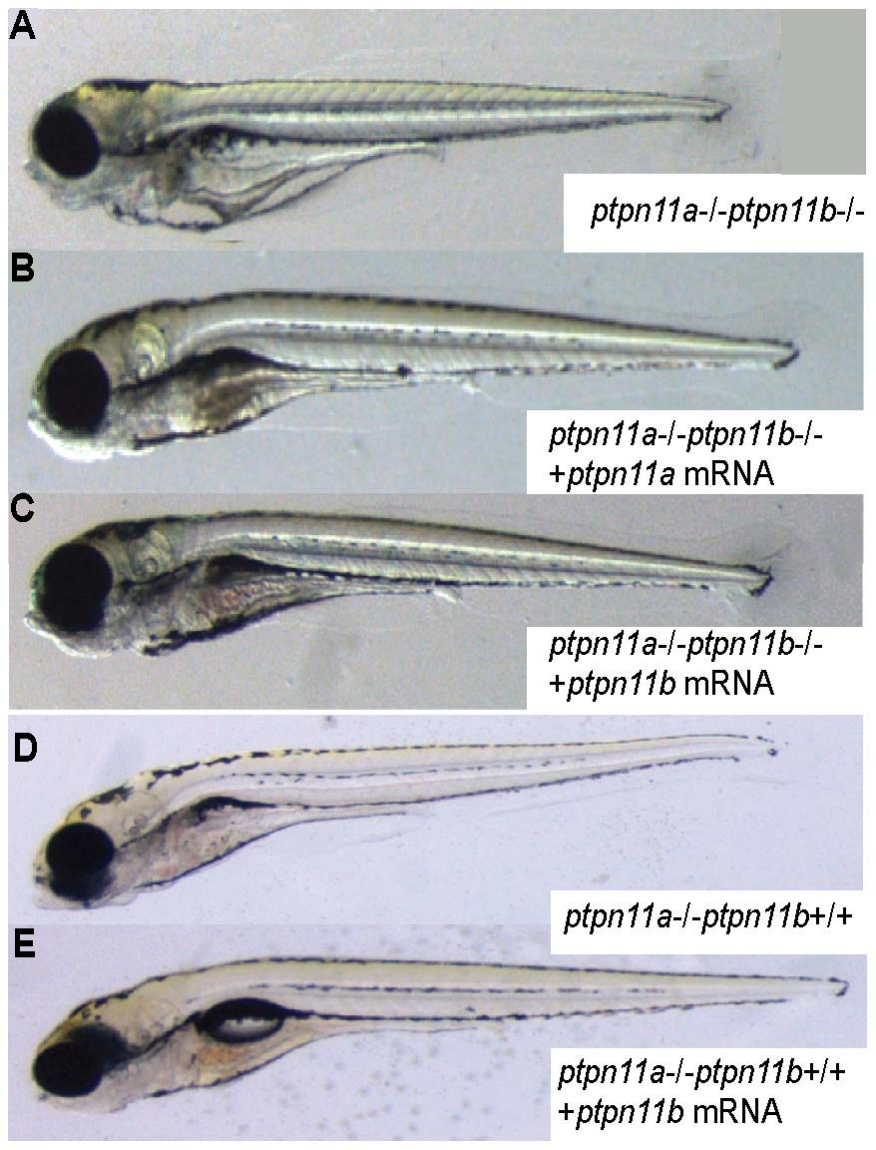
Rescue of mutant phenotype by exogenous *ptpn11a* and *ptpn11b*. Morphology of 5(a) *ptpn11a-/-ptpn11b-/-* double mutant, (b) double mutant expressing exogenous *ptpn11a,* (c) double mutant expressing exogenous *ptpn11b*, (d) *ptpn11a-/-* mutant, (e) *ptpn11a-/-* mutant expressing exogenous *ptpn11b*. Representative embryos are depicted here. See Table 2 for quantification.

To investigate if the double mutant phenotype could be suppressed by introduction of the full-length *ptpn11a* and *ptpn11b* mRNA, we performed rescue experiments. Clutches of embryos from *ptpn11a+/-ptpn11b-/-* zebrafish were split and injected with *ptpn11a* or *ptpn11b* mRNA, or were not injected. The embryos were sorted based on their morphology at 5 dpf and genotyped by sequencing. The overall morphology of double mutant embryos is nicely distinguishable from wildtype embryos and 40/199 - close to the expected 25% - of the embryos are shorter and particularly the trunk and tail region is wider (Fig. 6A). Microinjection of *ptpn11a* mRNA in offspring of *ptpn11a+/-ptpn11b-/-* mutants at the one-cell stage largely suppressed the morphological phenotype in double mutant embryos at 5 dpf (Fig.6B, Table 2). Interestingly, injection of *ptpn11b* mRNA also led to rescue of the morphological defects (Fig.6C, Table 2), indicating that *ptpn11a* and *ptpn11b* have redundant biochemical functions. The difference in the function of *ptpn11a* and *ptpn11b* in development may be caused by the observed difference in expression level at early stages of development. To test this hypothesis directly, we investigated whether *ptpn11b* RNA was able to rescue the *ptpn11a-/-ptpn11b+/+* phenotype. *Ptpn11b* mRNA injections at 1 cell stage indeed largely suppressed the *ptpn11a-/-* embryo phenotype (Fig.6D,E, Table 2). These results show that *ptpn11a* and *ptpn11b* mRNA injections largely rescued the developmental defects in the double mutant embryos at 5 dpf and that injections of *ptpn11b* alone suppressed the *ptpn11a-/-* phenotype.

**Table 2:** Rescue of *ptpn11a* mutant phenotype by injection of *ptpn11a* and *ptpn11b*.

incross	injection (mRNA)	phenotype	*ptpn11a-/-*/total
*ptpn11a+/-ptpn11b-/-*	-	40/199	40/199
*ptpn11a+/-ptpn11b-/-*	*ptpn11a*	1/98	23/98
*ptpn11a+/-ptpn11b-/-*	*ptpn11b*	5/65	12/65
			
*ptpn11a+/-ptpn11b+/+*	-	25/102	25/102
*ptpn11a+/-ptpn11b+/+*	*ptpn11b*	15/360	75/360

Clutches of embryos from *ptpn11a+/-ptpn11b-/-* or ptpn11a+/-ptpn11b+/+ zebrafish were split and injected with *ptpn11a* mRNA or *ptpn11b* mRNA. The embryos were scored based on their morphology at 5 dpf. Phenotype represents the number of embryos with mutant morphology/total number of embryos. Subsequently, the genotype was determined by sequencing and is depicted as number of *ptpn11a-/-* embryos/total number of embryos analyzed. *Ptpn11b* was homozygous (*ptpn11b-/-* or *ptpn11b+/+*) and was not determined in these embryos.

### Impaired Erk/MAPK, but not Akt/PKB signalling in Shp2 double mutant embryos

**Figure 7:**
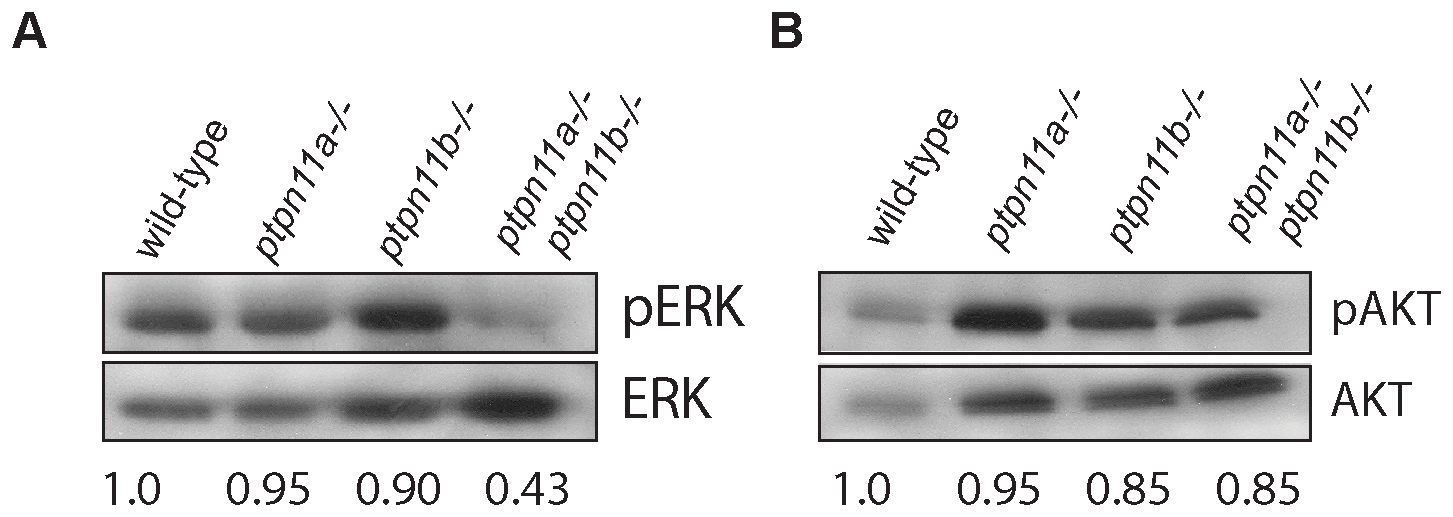
Impaired Erk/MAPK, but not Akt/PKB signalling in Shp2 double mutant embryos. WT, ptpn11a-/-, ptpn11b-/- and ptpn11a-/- ptpn11b-/- embryos were lyzed and immunoblotted with phosho-specific antibody for ERK and AKT; membranes were then reprobed for total Erk and Akt expression to control for loading. The blots were quantified and the ratio of phosphorylated protein/total protein from three independent experiments is indicated below each lane.

Shp2 is required to promote the activation of the MAPK cascade in response to growth factors, cytokine receptors and integrins [3], [36]. Thus, we examined the functional consequences of loss of Shp2 on downstream signaling by investigating Erk phosphorylation in wildtype, *ptpn11a-/-*, *ptpn11b-/-* and double mutant embryos at 5 dpf. Immunoblotting showed that Erk activation was decreased in double mutant embryos compared to wild type, whereas Erk levels were not impaired in the single homozygous mutants (Fig. 7A). Shp2 has been reported to act upstream of Akt/PKB signalling [12], [18]. We investigated Akt signaling by immunoblotting for phosphorylated Akt in wild-type, *ptpn11a-/-*, *ptpn11b-/-* and double mutant embryos at 5 dpf. We found that Akt activation was not affected in both single *ptpn11* homozygous mutants, nor in the double mutants (Fig. 7B). Together, these data indicate that Erk/MAPK signaling, but not Akt (PKB) signaling is affected in mutants lacking functional Shp2.

## Discussion

In this paper we characterized the two *ptpn11* genes in zebrafish, *ptpn11a* and *ptpn11b*. The products of these genes, Shp2a and Shp2b, respectively, are both active and exhibit comparable phosphatase activities. Using a TSGI approach, we identified nonsense mutations in *ptpn11a* and *ptpn11b*, well upstream of the catalytic site that abolished Shp2 function. In agreement with previous studies in mice with targeted *ptpn11* [20], [37], double mutant zebrafish embryos lacking all functional Shp2 were found to be embryonic lethal. Mouse embryos lacking functional Shp2 die around implantation [20], [37], whereas zebrafish embryos lacking functional Shp2 survive until 5-6 dpf. The difference in the developmental stage at which the lack of Shp2 is embryonic lethal between mice and zebrafish might be explained by the difference in maternal contribution of Shp2 in zebrafish eggs compared to mouse embryos.

Whereas double mutant zebrafish embryos are embryonic lethal, lack of *ptpn11b* in homozygous single mutant fish resulted in viable and fertile fish without developmental defects. Yet, *ptpn11a*-/- single mutant embryos did not survive, suggesting a distinct role of the two *ptpn11* genes in zebrafish development. Biochemically, Shp2a and Shp2b have similar catalytic activities. However, expression analyses showed that *ptpn11a* was abundant in all developmental stages whereas *ptpn11b* was hardly detectable at early stages. *Ptpn11b* expression increased strongly over time. This may explain why the lack of *ptpn11a* affected development and lack of *ptpn11b* did not. Low levels of *ptpn11b* in *ptpn11a-/-* embryos at early stages cannot compensate for the lack of *ptpn11a*. Conversely, high levels of *ptpn11a* in *ptpn11b-/-* embryos can compensate for the lack of *ptpn11b*. *Ptpn11a* as well as *ptpn11b* rescued developmental defects in double mutant embryos, indicating that Shp2a and Shp2b have redundant functions. It is noteworthy that expression of exogenous *ptpn11b* rescued *ptpn11a-/-* embryos, demonstrating that the difference in developmental function of *ptpn11a* and *ptpn11b* is mainly caused by differences in expression levels during early development.

The developmental defects we observed in *ptpn11a-/-ptpn11b-/-* embryos became apparent relatively late during development. Only at 4-5 dpf the first morphological defects were visible. This is in contrast with earlier reports of morpholino (MO)-mediated knockdown of Shp2, where we and others observed developmental defects as early as gastrulation [26], [27]. The lack of gastrulation defects in *ptpn11a-/-ptpn11b-/-* embryos may be due to maternal contribution of *ptpn11a* in the double mutants.

The reduced body axis and craniofacial defects we observed in double mutants are comparable to the phenotypes of Shp2 knockdown embryos and of embryos expressing NS or LS variants of Shp2, that we and others reported earlier [26], [27]. It is evident that Shp2 has an important role in body axis extension and craniofacial development: short stature is one of the most recognized symptoms in individuals with NS and LS. During the first years of life the body length drops below the 10th percentile for height in NS [38] and below the 25th percentile in LS [39]. Knock-in mice expressing Shp2 variants of NS or LS show postnatal growth retardation [40], [41]. Moreover, human patients with NS or LS show visible craniofacial symptoms, including high forehead, hypertelorism, downslanting palpebral fissures, epicantal folds, ptosis, low-set and/or posteriorly rotated ears [5], [42], [43]. The ablation of Shp2 in neural crest cell leads to severe craniofacial anomalies in mice [44], [45]. Similarly, expression of morpholino Shp2 and Noonan or LEOPARD Shp2 embryos, induced craniofacial defects in zebrafish at 4dpf [26], [27]. Collectively, our results showed craniofacial defects and body axis extension defects in mutant zebrafish embryos, lacking functional Shp2, that are consistent with defects observed in mouse models lacking functional Shp2 in relevant cell types as well as with developmental defects in mouse models for NS or LS and symptoms in human individuals with NS or LS.

SHP2 positively regulates (ERK)/MAPK signal transduction [2] and downregulation of the (ERK)/MAPK pathway, due to the disruption of Shp2, leads to the formation of skeletal abnormalities and skull defects in mice [46], [47].We show that at 5dpf, pERK levels are downregulated in *ptpn11a-/-ptpn11b-/-* embryos, which is consistent with an essential role for Shp2 in Erk/MAPK signaling upstream of craniofacial development. However, even though in the *ptpn11a-/-* embryos pERK levels are comparable to wildtype, these embryos still showed craniofacial defects and reduced body length at 5dpf, albeit to a lesser extent than *ptpn11a-/-ptpn11b-/-* double mutant embryos. We cannot exclude the possibility that craniofacial development is independent of Erk/MAPK signaling. However, the *ptpn11a-/-* phenotype may be the consequence of reduced Erk/MAPK signaling at earlier stages, i.e. prior to 5 dpf. *Ptpn11b* is only expressed at low levels at these early developmental stages, which may not compensate for the loss of *ptpn11a* to sustain physiological levels of Shp2-dependent Erk/MAPK signaling at stages that are crucial for craniofacial development. At 5 dpf, *ptpn11b* expression is high and may compensate for the lack of Shp2a in *ptpn11a-/-* mutants with respect to Erk/MAPK signaling. To prove temporal dependence on Erk/MAPK signaling in *ptpn11a-/-* mutant embryos is not trivial as it requires assessment of Erk phosphorylation at early developmental stages, i.e. in embryos that do not yet display developmental defects. Collectively, our data show that functional Shp2 is required for Erk/MAPK signaling at 5 dpf and whether Erk/MAPK signaling is involved in the developmental defects remains to be determined definitively.

In conclusion, we characterized the two *ptpn11* genes in zebrafish and found that the function of the two genes in development is distinct which is due to differences in their temporal expression patterns. The developmental defects that were observed in double mutant zebrafish embryos lacking functional Shp2 are consistent with developmental defects in mouse models lacking Shp2. The observed body axis extension and craniofacial defects are reminiscent of defects observed in mouse models for NS and LS, as well as the symptoms observed in human patients with NS and LS, underlining the importance of Shp2 for craniofacial development and body axis extension. The *ptpn11* mutants that we have identified constitute a useful system to further unravel the function of Shp2 in development. Moreover, these mutants provide a good starting point for the development of genetic models for NS and LS in zebrafish that will complement other animal models for these syndromes.

## Supporting Information

Figure S1 Comparison of human SHP2 and zebrafish Shp2a and Shp2b polypeptides. The alignment was done using ClustalW. Identical amino acids are highlighted in black. The consensus sequence is shown underlined, on top of the rows. Amino acids are numbered to the right of each row.
(PDF)
