# Supplementary material for: Distinct and Overlapping Functions of ptpn11 Genes in Zebrafish Development
Source: PLoS One. 2014 Apr 15;9(4):e94884. doi: 10.1371/journal.pone.0094884 (PMC3988099; doi:10.1371/journal.pone.0094884)
Supplement: Figure S1 — Comparison of human SHP2 and zebrafish Shp2a and Shp2b polypeptides. The alignment was done using ClustalW. Identical amino acids are highlighted in black. The consensus sequence is shown underlined, on top of the rows. Amino acids are numbered to the right of each row. (PDF) [file pone.0094884.s001.pdf]

```
hShp2.pro    MTSRRWFHPNITGVEAENLLLTRGVDGSFLARPSKSNPGDFTLSVRRNGAVTHIKIQNTGDYYDLYGGEKFATLAELVQYYMEHHGQLKEKNGDVIELKY  100
zshp2a.pro   MTSRRWFHPNITGVEAENLLLTRGVDGSFLARPSKSNPGDFTLSVRRNGAVTHIKIQNTGDYYDLYGGEKFATLAELVQYYMEHHGQLKEKNGDVIELKY  100
zshp2b.pro   MTSRRWFHPNITGIEAEHLLLTRGVHGSFLARPSKSNPGDFTLSVRRNDEVTHIKIQNSGDYYDLYGGEKFATLAELVQYYTEQHDLLRERNGDVIELKY  100

hShp2.pro    PLNCADPTSERWFHGHLSGKEAEKLLTEKGKHGSFLVRESQSHPGDFVLSVRTGDDKGESNDGKSKVTHVMIRCQ-ELKYDVGGGERFDSLTDLVEHYKK  199
zshp2a.pro   PLNCADPTSERWFHGHLSGREAEKLLTEKGKNGSFLVRESQSHPGDFVLSVRTGDDKTDTSDGKPKVTHVMIRCQHDLKYDVGGGEKFDSLTDLVEHYKK  200
zshp2b.pro   PLNCKDPTSERWYHGHLSGRDAEKLLTEKGKSGSFLVRESQSKPGDFVLSVLTNEEKHENVDRKTKVTHVMIRYQ-DGKYDVGGGERFDTLADLVEHYKK  199

hShp2.pro    NPMVETLGTVLQLKQPLNTTRINAAEIESRVRELSKLAETTDKVKQGFWEEFETLQQQECKLLYSRKEGQRQENKNKNRYKNILPFDHTRVVLHDGDPNE  299
zshp2a.pro   NPMVETLGTVLQLKQPLNTTRINAAEIESRVRELSKLAEATDKVKQGFWEEFETLQQQECKLLYSRKEGQRPENKNKNRYKNILPFDHTRVVLTDGDVNE  300
zshp2b.pro   NPMVEKSGIVVHLKQPFNATRINAANIENRVHELNKVADNSEKPKQGFWEEFEVLQQQECKLLYPRKEGQRPENKNKNRYKNILPFDTTRVQIKEADPDV  299

hShp2.pro    PVSDYINANIIMPEFETKCNNSKPKKSYIATQGCLQNTVNDFWRMVFQENSRVIVMTTKEVERGKSKCVKYWPDEYALKEYGVMRVRNVKESAAHDYTLR  399
zshp2a.pro   QGSDYINANLIMPDNEAKSNNSKLKRSYIATQGCLQNTISDFWRMVFQENSRVIVMTTKEVERGKSKCVKYWPDVSALKEYGAMRVRNVKETMAHDYILR  400
zshp2b.pro   PGSDYINANYIRSVNEEGRHMDE-GKVFIATQGCLQNTVLDFWKMVYQENTHVIVMTTKEMERGRNKCVRYWPDLNSTKEFGKVCVKNIEEHTAQDYIRR  398

hShp2.pro    ELKLSKVGQGNTERTVWQYHFRTWPDHGVPSDPGGVLDFLEEVHHKQESIMDAGPVVVHCSAGIGRTGTFIVIDILIDIIREKGVDCDIDVPKTIQMVRS  499
zshp2a.pro   ELKLSKVGQGNTERTVWQYHFRAWPDHGVPGDPGGVLDFLEEVKLKQEGITGAGPIVVHCSAGIGRTGTFIVIDILIDIIREKGVDCDIDVPKTIQMVRS  500
zshp2b.pro   ELEVTRLDRREPPRCIWHYQYLSWPDHGVPNEPGGVLSFLEQVNRTQSAIPESGPIVVHCSAGIGRTGTIIVIDILIDIINRQGLDCDIDIPKTIQRVRQ  498

hShp2.pro    QRSGMVQTEAQYRFIYMAVQHYIETLQRRIEEEQKSKRKGHEYTNIKYSLADQTSGDQSPLPPCTPTPPCAEMREDSARVYENVGLMQQQKSFR        594
zshp2a.pro   QRSGMVQTEAQYRFIYMAVQHYIETLQRRIEEEQKSKIKGREYTNIKYSLSDLSGGDQSPLPPCTPTPTCADMRDDSSRVYENVGLMQQQKSHR        594
zshp2b.pro   QRSGMVQTEAQYKFIYMAVQQYIDTAQKRLEEEQRNKTKEREYSNIKYPQMSN-AR---AKPNMSSSRSSSVMNDDSS-VYENLNIKNPKGSTSSNTRR  592
```
